# Can calcitonin gene‐related peptide monoclonal antibodies ameliorate writer's cramp and migraine?

**DOI:** 10.1002/npr2.12444

**Published:** 2024-04-11

**Authors:** Keisuke Suzuki, Shiho Suzuki, Hiroaki Fujita, Hirotaka Sakuramoto, Mukuto Shioda, Koichi Hirata

**Affiliations:** ^1^ Department of Neurology Dokkyo Medical University Mibu Japan

**Keywords:** calcitonin gene‐related peptide, focal task‐specific dystonia, migraine

## Abstract

Recently, calcitonin gene‐related peptide (CGRP) monoclonal antibodies (mAbs) have become available as a prophylactic treatment for migraine and have shown high efficacy and safety in clinical practice. CGRP mAbs have been reported to be effective not only for migraine but also for other comorbidities, such as psychiatric complications in patients with migraine. However, there are no reports examining the effect of CGRP mAbs on dystonia. We treated a patient with comorbid migraine and focal task‐specific dystonia (writer's cramp) with a CGRP mAb (erenumab) because of an increase in monthly migraine days despite the addition of migraine prophylaxis. In this patient, erenumab treatment for 3 months led to improvements in symptoms of both focal dystonia and migraine, suggesting a role for CGRP in the pathophysiology of both conditions.

## Abstract 2

Calcitonin gene‐related peptide (CGRP) monoclonal antibodies (mAbs) have become available as a prophylactic treatment for migraine. We treated a patient with comorbid migraine and focal dystonia with a CGRP mAb (erenumab). In our patient, erenumab treatment for 3 months led to improvements in symptoms of both focal dystonia and migraine.

## Graphical abstract

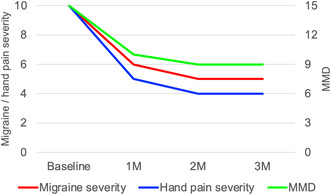

## 1 INTRODUCTION

Migraine is a common highly disabling neurologic disorder, and in recent years, effective migraine‐specific prophylaxis has become available. Calcitonin gene‐related peptide (CGRP) monoclonal antibodies (mAbs), such as erenumab, fremanezumab, and galcanezumab, are potent migraine prophylactic agents that target CGRP, a vasodilator that is released from trigeminal nerve endings during migraine attacks and is involved in migraine pathogenesis.^1^ In Japan, CGRP mAbs are currently available for use in migraine patients with four or more migraine days per month, despite the use of at least one conventional migraine prophylactic drug, or when conventional prophylactic drugs cannot be used due to concerns about side effects. CGRP mAbs have also been reported to be effective at treating coexisting restless legs syndrome^2^ and psychiatric complications such as anxiety and depression^3^ in patients with migraine. Dystonia is a movement disorder characterized by sustained or intermittent muscle contractions that cause abnormal, repetitive movements and postures often accompanied by pain. Here, we report the case of a patient with combined migraine and task‐specific focal dystonia who was successfully treated with a CGRP mAb.

## 2 CASE REPORT

**FIGURE 1:**
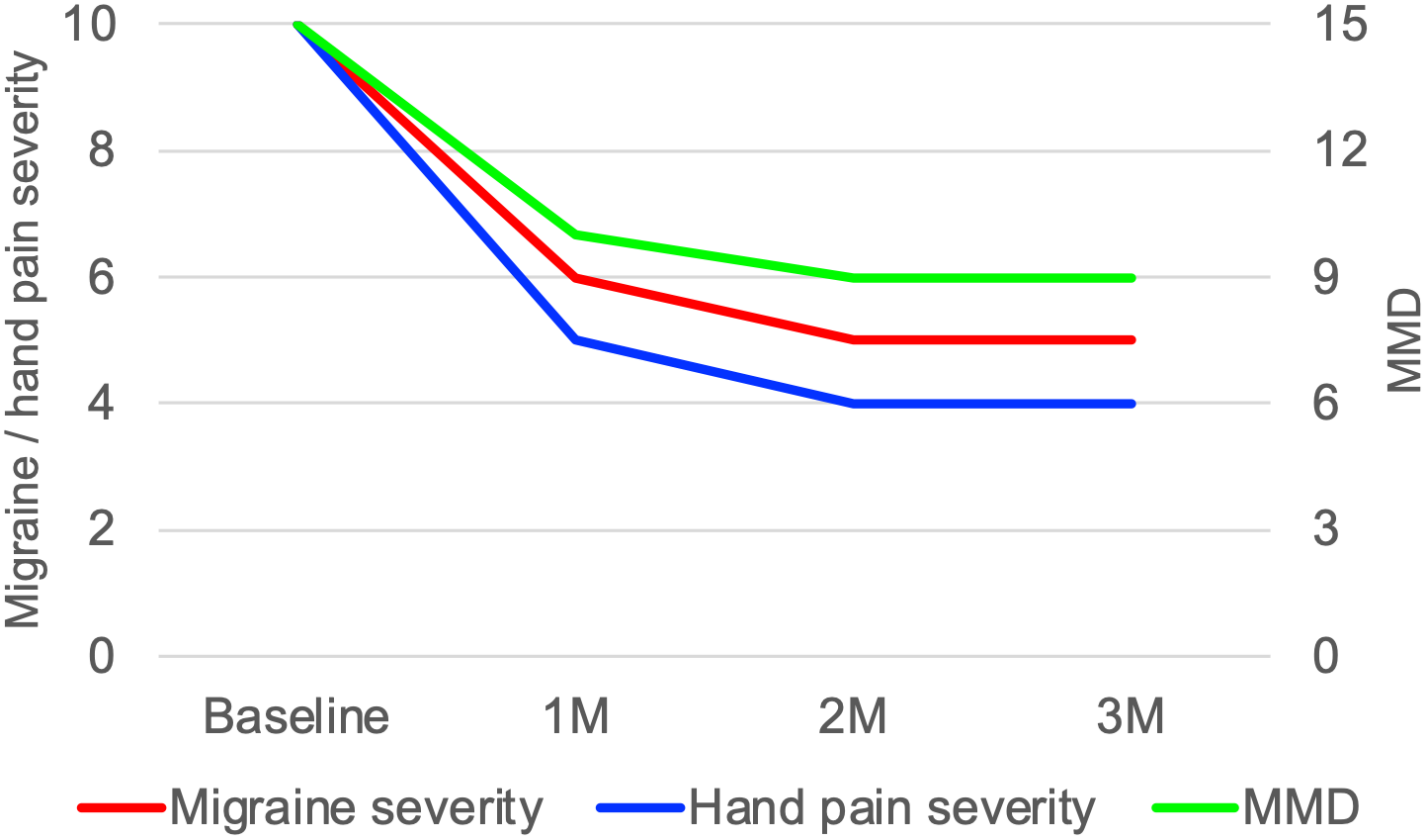
Clinical course of the patient after treatment with erenumab. MMD, monthly migraine days; M, months. Hand pain and headache severity (0–10) are shown on the left vertical axis, and MMD is shown on the right vertical axis.

A 42‐year‐old woman presented with complaints of difficulty writing due to involuntary movements of her right hand when writing. She had been writing documents every day at her office job. Since the age of 41 years, she had difficulty writing due to the extension of her right wrist and right fingers. The symptoms initially appeared approximately three times a week when she wrote for more than 30 min, but after 1 month, they began to appear every day, even when she wrote for only a few minutes. She often had to stop writing because of hand pain. No right‐hand symptoms occurred except when writing. On neurologic examination, the patient showed no motor weakness or parkinsonism. Brain magnetic resonance imaging (MRI) revealed no obvious abnormalities. A dopamine transporter scan showed no decrease in striatal 123 I‐ioflupane accumulation. The patient was diagnosed with focal task‐specific dystonia (writer's cramp) since symptoms appeared exclusively when the patient was writing. The patient's symptoms were alleviated by 6 mg of trihexyphenidyl and 0.5 mg of clonazepam, but she still experienced pain in her right hand when writing. The patient did not wish to be treated with botulinum toxin. The patient had experienced migraine without aura since her late 30s. Her migraine was often accompanied by photophobia and nausea, and she recently experienced an increase in the number of headache days. The beta‐blocker propranolol (30 mg/day) and calcium channel blocker lomerizine (10 mg/day) were administered for 2 months each as migraine prophylaxis, but no significant effect was observed. After the failure of two types of prophylactic treatment for migraine, treatment with 70 mg of erenumab, a CGRP mAb, was initiated. After 1 month of erenumab treatment, the number of migraine days per month decreased from 15 to 10 days, and the severity of migraine and right‐hand pain during writing decreased. The patient no longer needed to take topical or oral nonsteroidal anti‐inflammatories when writing. Figure 1 illustrates the 3‐month treatment course. The severity of right‐hand pain and migraine were rated on a 0–10 scale. Improvements in migraine and hand symptoms were observed over 3 months after starting treatment with erenumab. Thereafter, the patient was introduced to self‐injection at home and was scheduled to receive a follow‐up visit 2 months later.

## 3 DISCUSSION

Our patient showed improvements in migraine and writer's cramp symptoms over 3 months after starting treatment with erenumab. The American Headache Society states that the efficacy of CGRP mAbs should be evaluated after 3 months, and treatment should be continued only if there is a 50% or greater reduction in migraine days per month compared to baseline or if there is a clinically meaningful decrease in the disability score.^4^ Regarding how long to continue CGRP mAbs, in patients with episodic or chronic migraine, the European Headache Federation states that discontinuation of a CGRP mAb should be considered after 6–12 months.^5^ In Japan, although there are no clear restrictions on the continuation of CGRP mAb use, the Japanese CGRP‐related new migraine treatment guideline (tentative version) states that the therapeutic benefit should be evaluated after 3 months, and if symptoms do not improve, the discontinuation of CGRP mAb should be considered, followed by regular reviews of treatment efficacy.^6^ In our patient, the reduction in monthly migraine days did not reach more than 50% after 3 months, but there was a clinically significant decrease in migraine severity. Therefore, we decided to continue treatment with erenumab, and we plan to conduct a regular review of treatment efficacy at each future follow‐up visit.

The pathophysiology of focal task‐specific dystonia has been linked to the involvement of the entire sensorimotor network and aberrant basal ganglia function.^7^ In animal studies, the presence of CGRP or CGRP receptors has been confirmed in multiple brain regions, including the cerebellum, hippocampus, hypothalamus, amygdala, and basal ganglia, in addition to the trigeminal nucleus caudalis,^8^ and the colocalization of dopamine and CGRP has also been reported in the hypothalamic A11 region.^9^ CGRP mAbs exert their therapeutic effects mainly on the trigeminal ganglion and trigeminal neurons innervating the dura mater, and a very small percentage of CGRP mAbs cross the blood–brain barrier^10^; thus, the central nervous system is unlikely to be the primary site of action. However, after treating patients with migraine with erenumab, the activation of the hypothalamus on functional brain MRI was reduced, suggesting central effects of CGRP mAbs.^11^ Cortical spreading depolarization induces CGRP release in the cortex and from trigeminal afferents in the meninges, and the binding of CGRP to receptors on the peripheral terminals of trigeminal Aδ fibers leads to the activation of neurons within the trigeminal cervical complex via the release of glutamate.^12^ It has been proposed that peripheral CGRP antagonism may reduce cortical glutamate levels and migraine aura. Therefore, the central effects of CGRP mAbs may have had an ameliorative effect on writer's cramp in this patient.

Botulinum toxin is used as a treatment for focal dystonia, although this treatment was not attempted in our patient.^13^ The mechanism of action of botulinum toxin includes blocking cholinergic neuromuscular release, as well as inhibiting the synaptic release of excitatory neurotransmitters such as acetylcholine, glutamate, CGRP, and substance P.^14^ Onabotulinum toxin A has been approved for the treatment of chronic migraine. The mechanisms by which onabotulinum toxin A ameliorates migraine include increasing the threshold for nociceptive activation by decreasing circulating levels of neuropeptides such as CGRP, decreasing the number of TRPV1‐immunoreactive neurons in the trigeminal ganglia, decreasing dorsal horn neuron activation, and decreasing the expression of nitric oxide synthase in the central nervous system.^7^ Therefore, although there are no reports directly examining the effect of CGRP antagonists on dystonia, CGRP antagonism may be beneficial for reducing not only migraine but also focal dystonia, considering the mechanism of action of botulinum toxin mediated by CGRP. However, further studies on the effects of CGRP antagonism on migraine and its comorbidities are needed.

## AUTHOR CONTRIBUTIONS

KS, SS, HF, and KH were involved in the conception and design of this study. KS wrote the first draft of this article. SS, HF, HS, MS, and KH contributed to the revision of this article. All the authors have read and approved this final article.

## FUNDING INFORMATION

The authors received no financial support for the research, authorship, or publication of this article.

## CONFLICT OF INTEREST STATEMENT

The authors declare the following potential conflicts of interest with respect to the research, authorship, and/or publication of this article: K Suzuki received lecture fees from Eli Lilly Japan, Daiichi Sankyo, and Otsuka Pharmaceutical Co. Ltd., outside of the submitted work. S Suzuki received lecture fees from Eli Lilly Japan, Daiichi Sankyo, Amgen and Otsuka Pharmaceutical Co. Ltd., outside of the submitted work. H Fujita, H Sakuramoto, and M Shioda had nothing to disclose. K Hirata received lecture fees from Eli Lilly Japan, Daiichi Sankyo, Amgen and Otsuka Pharmaceutical Co., Ltd., outside of the submitted work.

## ETHICS STATEMENT

Approval of the Research Protocol by an Institutional Review Board: N/A.

Informed Consent: N/A.

Registry and the Registration No. of the Study/Trial: N/A.

Animal Studies: N/A.

## Data Availability

The relevant data are contained within this article.
